# Telehealth for all: Rwanda's digital leap in sexual and reproductive healthcare access

**DOI:** 10.1080/26410397.2026.2709970

**Published:** 2026-07-29

**Authors:** Frida Temple, Muhammed Semakula, Thierry Sebakunzi, Marie Claire Iryanyawera, Olugbemiga Adelakin, Renata Tallarico

**Affiliations:** a SRHR and Emerging Trends Specialist, United Nations Population Fund, Kigali, Rwanda.; b Permanent Secretary, Ministry of Health, Department of Planning, Strategic Information and Health Financing, Kigali, Rwanda; c Officer, Ministry of Health, Department of Planning, Strategic Information and Health Financing, Kigali, Rwanda; d SRHR Team Lead, United Nations Population Fund, Kigali, Rwanda; e Country Representative, United Nations Population Fund, Kigali, Rwanda; f Deputy Representative and Head of Programs, United Nations Population Fund, Kigali, Rwanda

**Keywords:** telehealth, comprehensive abortion care, sexual and reproductive health and rights, global health, innovation, Rwanda

## Abstract

In Rwanda, unsafe abortion contributes significantly to maternal mortality despite a legal framework permitting abortion under specific circumstances. Barriers to accessing safe abortion services persist, necessitating innovative solutions. This commentary describes the development and implementation of a telehealth tool for the provision of safe abortion care in Rwanda, driven by national ownership and youth engagement. Framed within a human rights-based approach to health equity, this initiative saw young Rwandan innovators create a cost-effective and context-fit digital solution through a hackathon modality. The hackathon process led to the transition of the telehealth tool, which was being piloted, from fragmented, informal communication channels into a secure platform that ensured data privacy, patient confidentiality and standardised clinical documentation. The new platform facilitates remote consultation between the patient, the midwife/nurse and the medical doctor, while providing support to healthcare providers. This initiative demonstrates the potential of leveraging local talents and the power of home-grown solutions in the area of digital technology to improve access to essential healthcare services, bridging the gap between rural primary care and specialised medical oversight. Ultimately, this contributes to the reduction of maternal morbidity and mortality in Rwanda. Moreover, the paper underscores the need for a continuous evaluation of telehealth effectiveness to ensure ethical, rights-based and legal alignment. The project's success underscores the importance of a needs-driven and people-centred approach, government ownership and the active role of young people as co-designers in developing and implementing sustainable digital health solutions.

## Résumé

Au Rwanda, les avortements non médicalisés contribuent sensiblement à la mortalité maternelle, en dépit d’un cadre juridique qui autorise l’avortement dans certaines circonstances. Des obstacles persistent à l’accès aux services d’avortement sans risque, exigeant des solutions innovantes. Ce commentaire décrit l’élaboration et la mise en œuvre d’un outil de télésanté pour l’octroi de soins d’avortement sans risque au Rwanda, grâce à une appropriation nationale et à l’engagement des jeunes. S’inscrivant dans une approche de l’équité en santé fondée sur les droits humains, cette initiative a permis à de jeunes innovateurs rwandais de créer une solution numérique rentable et adaptée au contexte par le biais d’un hackathon. Ce processus de hackathon a permis de faire évoluer l’outil de télésanté, alors en phase pilote, de canaux de communication informels et fragmentés vers une plateforme sécurisée garantissant la protection des données, la confidentialité des patientes et la normalisation de la documentation clinique. La nouvelle plateforme facilite la téléconsultation entre la patiente, la sage-femme/l’infirmière et le médecin, tout en apportant un soutien aux professionnels de santé. Cette initiative démontre le potentiel de mobilisation des talents locaux et la force de solutions imaginées localement dans le domaine de la technologie numérique pour améliorer l’accès aux services de santé essentiels, comblant l’écart entre les soins primaires en milieu rural et la supervision médicale spécialisée. En définitive, cela contribue à la réduction de la morbidité et de la mortalité maternelles au Rwanda. De plus, l’article souligne la nécessité d’une évaluation continue de l’efficacité de la télésanté pour garantir sa conformité aux principes éthiques, juridiques et fondés sur les droits. Le succès du projet met en lumière l’importance d’une approche basée sur les besoins et axée sur les personnes, de l’appropriation par les autorités et du rôle actif des jeunes en tant que coconcepteurs dans la création et l’application de solutions de santé numérique durables.

## Resumen

En Ruanda, el aborto inseguro contribuye de manera significativa a la mortalidad materna a pesar de existir un marco legislativo que permite el aborto bajo circunstancias específicas. Las barreras al acceso a servicios de aborto seguro persisten, por lo que se necesitan soluciones innovadoras. Este comentario describe la creación y aplicación de una herramienta de telesalud para la prestación de servicios de aborto seguro en Ruanda, impulsada por apropiación nacional y la participación de jóvenes. Planteada como un enfoque de equidad de salud basado en los derechos humanos, esta iniciativa vio a jóvenes ruandeses innovadores crear una solución digital rentable y adecuada para el contexto por medio de una modalidad de hackathon. El proceso hackathon llevó a la transición de la herramienta de telesalud, que estaba siendo piloteada, desde canales de comunicación fragmentados e informales a una plataforma segura que garantizaba privacidad de datos, confidencialidad de pacientes y documentación clínica normalizada. La nueva plataforma facilita la consulta remota entre la paciente, la partera/enfermera y el médico, a la vez que brinda apoyo a profesionales de salud. Esta iniciativa demuestra el potencial de aprovechar los talentos locales y el poder de soluciones locales en el área de tecnología digital para mejorar el acceso a servicios de salud esenciales, cerrando la brecha entre la atención primaria rural y la supervisión médica especializada. A la larga, esto contribuye a la reducción de la morbimortalidad materna en Ruanda. Además, el artículo subraya la necesidad de una evaluación continua de la eficacia de la telesalud para garantizar una alineación ética, basada en los derechos y legislativa. El éxito del proyecto recalca la importancia de un enfoque impulsado por necesidades y centrado en las personas, apropiación gubernamental y la participación activa de jóvenes como codiseñadores en la creación y aplicación de soluciones de salud digital sostenibles.

## Introduction

Over the past 20 years, Rwanda reduced maternal mortality from 1071 to 203 per 100,000 live births.^1^ However, the decline has significantly slowed down in the last five years. This stagnation raises critical concerns about gaps in policy execution and the need for innovative interventions. Ten to seventeen percent of maternal mortality in Rwanda is related to abortions.^2^ This shows the need to improve access to and quality of abortion care to the full extent of the law in Rwanda.

Complications from unsafe abortion pose a significant public health challenge in Rwanda. Based on results from a national-level study conducted by Dusinga et al, which included data from 165 facilities in Rwanda, the rate of abortion complications at the health facility level was estimated at 10.7 per 1000 women aged 15–44 years old.^3^ While progress has been made, maternal mortality remains high, as evidenced by the confidential inquiry into maternal death, which estimated that 12% of maternal deaths in 48 public hospitals in 2019 were related to abortion.^4^ A study in rural northern Rwanda found that limited access to sexual and reproductive health (SRH) services is a factor influencing women towards unsafe abortions. The study recommends increasing access to quality youth-friendly SRH services and expanding safe abortion services in rural areas.^5^

Beyond being a public health necessity, access to safe abortion is a human rights issue, recognised by the World Health Organization, which advocates for a human rights-based approach to reproductive care.^6^ In Rwanda, the legal framework is grounded in the country’s commitment to the Maputo Protocol, ratified in 2005, which recognises reproductive health as a prerequisite for women’s equality and bodily autonomy.^7^ The persistent gaps in access to safe services represent a barrier to the right to health for many women, particularly those in underserved rural areas.

To address these preventable deaths, the Government of Rwanda has established a supportive policy framework to accelerate progress, including the integrated Reproductive, Maternal, Newborn, Child and Adolescent Health (RMNCAH) Policy 2018–2030 and the Family Planning/Adolescent Sexual and Reproductive Health (FP/ASRH) strategic plan 2018–2024.^8,9^ In addition, the Government of Rwanda has established a clear legal and guidance framework to address abortion-related mortality. Under current national legislation,^10^ abortion is exempt from criminal liability under the following specific circumstances:

1. The pregnant person is a child;
2. The pregnancy is a result of rape;
3. The pregnancy resulted from a forced marriage;
4. The pregnancy resulted from incest up to the second degree;
5. The pregnancy poses a risk to the health of the pregnant person or the fetus.

To facilitate the clinical implementation of these provisions, the Ministry of Health released a comprehensive Guidance Document to Operationalize the Exemptions for Abortion.^10^ Furthermore, a specific Ministerial Order was published to determine the conditions required for medical doctors to perform these procedures within a recognised healthcare setting.^11^ Despite these legal advancements, challenges persist in ensuring that these exemptions translate into accessible, stigma-free, and high-quality care for women in need.

In 2023–2024, the Termination of Pregnancy (TOP) guideline and training manuals were developed as a way to align the current Comprehensive Abortion Care guideline with the new WHO guideline for abortion, ensuring that safe abortion can be provided to the full extent of the law in Rwanda.^12^ Consistent uptake of quality sexual and reproductive health services requires new ways of working, including digital tools for telehealth.^13^ Decentralising primary health care ensures larger uptake, increases cost-effectiveness and reduces barriers to accessibility. Telehealth services offer a strategic pathway to overcome these existing barriers by decentralising primary health care. In Rwanda’s decentralised health system, primary care is delivered at the health centre level by nurses and midwives, while the legal medical authorisations required for abortion are typically reserved for district hospitals where medical doctors are stationed. Telehealth bridges this gap, allowing rural providers to consult with doctors instantaneously. The potential for this approach was demonstrated by an operational research study in the Musanze district, where comprehensive abortion care was provided in health centres by midwives and nurses under the remote oversight of medical doctors via telehealth. The research showed very good results, which led to the scale-up of the research to an additional five districts. However, the implementation strategy of the telehealth tool showed the need to improve safety and confidentiality.^14^

Specifically, the initial pilot relied on informal digital communication channels, including email, WhatsApp and Zoom. While these facilitated remote consultation, they lacked structured data entry and the robust encryption necessary to guarantee patient privacy and legal security. In collaboration with the Ministry of Health, UNFPA supported the development of an improved telehealth tool aiming at addressing these challenges in August 2024. This paper aims to document the development process of a telehealth tool via a hackathon for young innovators, highlighting how this collaborative approach can create secure, youth-led digital solutions for sexual and reproductive health and rights (SRHR) within the Rwandan context and beyond.

## The process of developing a telehealth tool

Initially, a meeting was held in May 2024, with relevant stakeholders, including clinical services officers from the Ministry of Health, representatives from the Rwanda Biomedical Centre, and frontline healthcare providers (midwives, nurses and doctors) from health centres and district hospitals, to assess the specific needs of patients and health providers to inform the development of the tool. The assessment confirmed the need for a digital tool and stressed the importance of designing a solution centred on ensuring data privacy, providing a standardised clinical checklist and creating a seamless user interface for low-bandwidth environments that was aligned with Rwanda's broader health system infrastructure. The objective was to transition from informal, fragmented communication to a unified, purpose-built platform.

During consecutive consultative meetings with Information and Communication Technology (ICT) experts, healthcare providers, and technical experts, the Terms of Reference (ToR) for the digital tool were developed. These discussions established a clear development plan for the hackathon centred on four critical functional requirements: the provision of asynchronous and synchronous consultation capabilities via chat and video; the integration of automated encryption protocols compliant with Rwanda’s Data Protection Law; the assurance of compatibility with Ministry of Health (MoH) central servers to maintain national data ownership and the implementation of role-based access control to provide distinct, secure interfaces for providers, doctors and administrators.

This was followed by preparatory meetings for the tool development, where the Ministry of Health decided to opt for a hackathon modality. This is a competitive, intensive design event where multidisciplinary teams collaborate to solve a specific problem within a short timeframe. This method was chosen over traditional procurement to foster home-grown innovation, ensure cost-effectiveness, allow for rapid prototyping and harness the innovation of young Rwandan professionals – specifically, software developers.

An advertisement was published for teams or individuals with specified digital and technical experience to apply to be part of the hackathon. Out of 70 applications, 7 teams were selected to join the hackathon.

The hackathon was held over a 9-day period during which the teams were hosted in the Digital Health hub in a facility in Kigali. The teams were provided with informative sessions about the use of the telehealth tool, the legal implications of comprehensive abortion care services and the need for the tool to be compatible with existing systems at the Ministry of Health. Midway through the hackathon, a team from the Ministry of Health and Rwanda Biomedical Center (RBC), the implementing arm of the Ministry of Health, reviewed the progress with daily close follow-ups. The hackathon concluded with all teams presenting their tools to a jury of experts from the Ministry of Health, Rwanda Biomedical Centre, Rwanda Health Initiative for Women and Youth, and UNFPA. The first, second and third prizes were awarded in a ceremony where the winning tool was presented.

After the hackathon, the winning teams collaborated to finalise the tool. They met with technical experts, including senior software architects from the Rwanda Biomedical Centre and MoH and clinical informatics specialists, to review its user-friendliness and ensure the language used aligned with the national technical standards. These standards refer to the MoH requirements for data interoperability, security and the use of approved clinical terminologies within the Rwandan digital health ecosystem.

**Figure 1.**
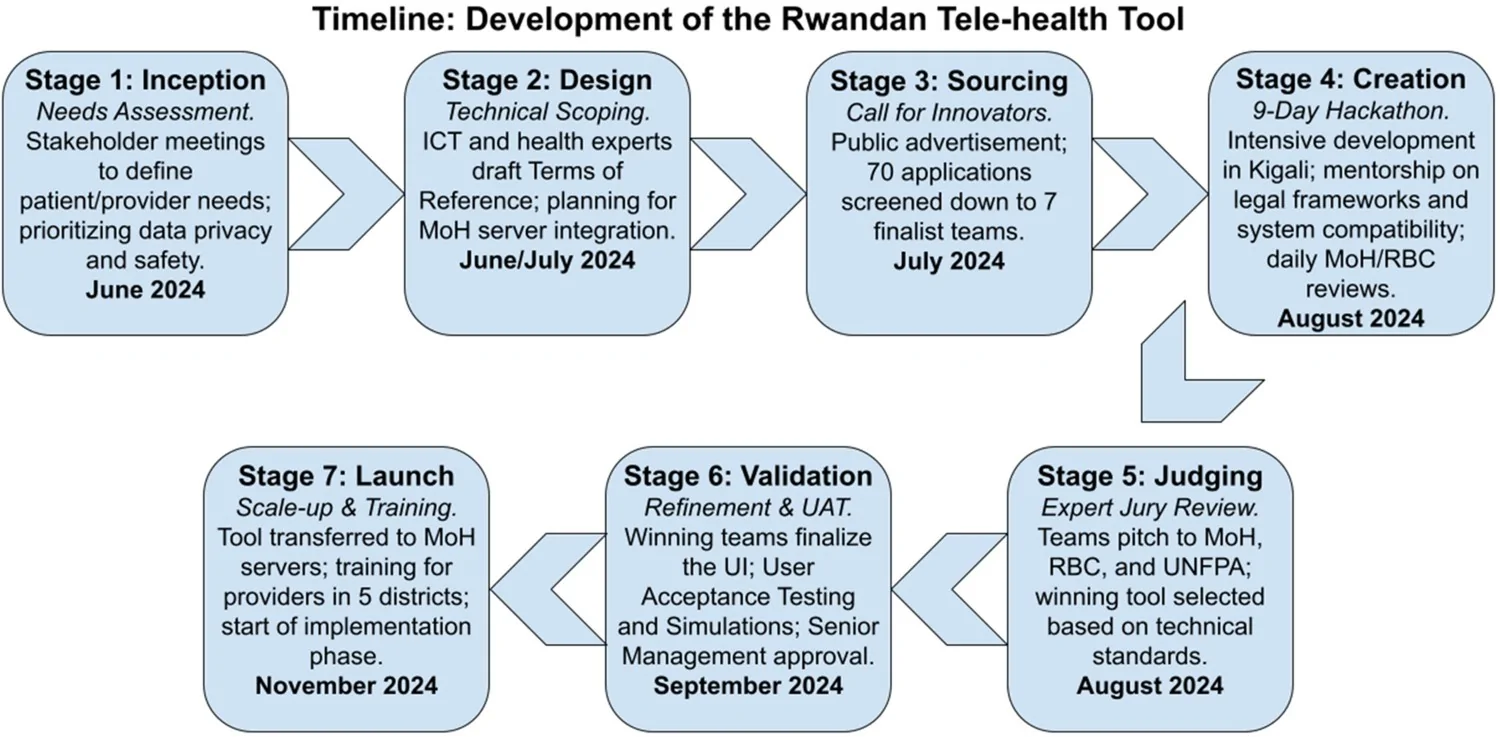
The development stages and timeline

The tool was then revised accordingly. Following this, a meeting with the head of the digital division at the Ministry of Health determined the date for User Acceptance Testing (UAT) and Simulation. Three weeks later, the tool was presented, tested via Simulation and UAT, and subsequently approved by the senior management at the Ministry of Health. Finally, the tool was transferred to the servers at the Ministry of Health. See Figure 1 for development stages and timeline.

### Tool architecture and data security

The developed telehealth platform operates as a secure, web-based application optimised for both desktop and mobile devices used in health centres. Its architecture comprises three primary modules:

- **The provider interface:** Allows midwives and nurses to input patient history via standardised clinical checklists, upload relevant ultrasound or laboratory findings, and request a virtual consultation with a medical doctor in the district hospital.
- **The doctor portal:** Enables medical doctors to review cases remotely, conduct video consultations and provide the mandatory legal endorsement for the procedure via digital signature, centralising the legal authorisation process.
- **The administrative dashboard:** Accessible only to MoH/RBC coordinators for real-time monitoring of service uptake and system performance, ensuring data are anonymised.

To address the confidentiality challenges identified in the pilot, the tool employs end-to-end encryption for all patient–provider communications. No patient-identifiable data is stored locally, but transmitted directly to and encrypted on the Ministry of Health’s secure servers, compliant with Rwanda’s Data Protection and Privacy Law.

### Implementation and deployment

The implementation of the tool was conducted in four districts in Rwanda (namely, Musanze, Ruhango, Karongi, and Rwamagana), and followed a structured deployment protocol:

1. Verification of internet connectivity and hardware at the participating health centres across the four districts to ensure readiness.
2. Before seeing actual patients, providers underwent “mock consultations” using the tool to ensure that they were comfortable with the interface and the functions.
3. During the initial months, technical leads from the winning hackathon team were working in the Ministry of Health and were able to provide physical or virtual support to clinicians.
4. Monthly virtual debriefs were held between health centre staff and the Ministry of Health to troubleshoot technical bugs and refine the user interface in real-time.

This rollout included specialised training for providers and investigators to ensure that the digital tool was effectively integrated into the existing referral pathways between health centres and district hospitals.

## How national ownership and young people's talent lead change

National ownership was pivotal to the development process. The government, through the Ministry of Health and Rwanda Biomedical Centre, acted as the primary architect of the project, defining the clinical requirements and providing the secure server infrastructure. This was a locally-led initiative in that the entire design and development cycle was powered by Rwanda, harnessing the talent of young people, ensuring that the tool met the country’s needs and priorities. This initiative was directly spearheaded by the authors within the Ministry of Health and Rwanda Biomedical Centre, who acted as both technical mentors and institutional leads to ensure the tool's alignment with national health strategies. This locally-led approach offered several advantages:

- **The needs-driven development** led to the immediate implementation of the tool. This sparked an interest in the young people not only to develop a tool, but to truly be a part of enhancing the country's health system. By embedding the development process within the Ministry’s own digital health division, the transition from prototype to public health infrastructure was seamless.
- **The government ownership** ensured the swift implementation and the immediate transfer of the tool to the MoH servers. As per the pre-hackathon agreement, the Ministry of Health retained all rights to the tool.
- **Cost-effectiveness:** The hackathon model proved to be more cost-effective than traditional software procurement. By utilising the government-owned Digital Health Hub for the hackathon and engaging young local innovators, the project bypassed the high costs associated with international procurement and external technical consultants. Preliminary calculations suggest that the total expenditure for the 9-day hackathon and subsequent refinement and implementation was approximately 60% lower than the projected cost of a standard 12-month software development contract with a private firm, while simultaneously achieving a faster “proof-of-concept” for deployment.
- **Empowerment and recognition of national talent:** The use of young national talent for this initiative allowed the government to further appreciate the potential of young people and their contribution towards development. The Ministry of Health expressed interest in retaining the team to support further digital transformation initiatives.

### Challenges faced and next steps

While the initial pilot demonstrates a strong proof-of-concept, full national integration presents potential challenges. Primarily, the platform may encounter stigma surrounding comprehensive abortion care among both communities and healthcare providers, which could potentially influence service utilisation and delivery. Furthermore, scaling the tool nationally introduces potential operational complexities, including variations in digital infrastructure alongside continuous workforce training to maintain data security compliance. These challenges are being addressed through training and national digitalisation.

The immediate next steps involve evaluating the current pilot in the five districts to test its efficacy and ensure the system can be scaled efficiently. Once the pilot achieves established benchmarks for safety and usability, the platform will be scaled up nationally. The next steps are to pilot this solution in the five districts to test its efficacy and ensure it can be used efficiently at a larger scale. Once this is established, the tool will be scaled up nationally. Furthermore, the tool is designed for modular expansion to include additional healthcare services, such as prenatal monitoring, family planning consultations, post-partum haemorrhage, emergency obstetric care and psychological support, thereby creating a virtual hospital with specialised support accessible to all areas.

## Conclusion

In conclusion, the development and implementation of this telehealth tool demonstrates the power of national ownership and youth engagement in addressing health gaps and advancing health equity. Evidence shows that telehealth interventions produce positive outcomes when used for remote patient monitoring, improving quality of life and reducing mortality.^15^ By bridging the gap between rural health centres and specialised medical doctors, the tool directly addresses the systemic inequities that often prevent women from exercising their reproductive rights. Advancing rights requires more than legal reform; it requires the creation of safe, accessible pathways for care. By utilising local young innovators, Rwanda has gained a home-grown, cost-effective digital solution that is perfectly appropriate for its specific context.

This project aligns directly with the Second Rwanda National Strategy for Transformation,^16^ which prioritises the transition to a knowledge-based economy through digital innovation, and the Fifth Health Sector Strategic Plan,^17^ which aims to leverage technology to achieve universal health coverage.

While this specific digital platform is newly deployed, early evidence from the precursor pilot in Musanze district indicated that 98% of consultations were successfully completed with high levels of provider satisfaction and no adverse clinical outcomes recorded during the research period.^14^ This successful proof-of-concept suggests the new, secured tool has the potential to significantly improve access to safe abortion services. Further developing the concepts of this study will involve a rigorous evaluation to measure the tool's impact on clinical outcomes and user satisfaction. Ultimately, this journey provides a scalable framework for integrating innovation led by young people into the broader landscape of essential healthcare services in Rwanda and beyond. This model directly contributes to Sustainable Development Goal 3 (Good Health and Well-being) and Goal 5 (Gender Equality),^18^ providing a replicable blueprint for other low- and middle-income countries seeking to use technology to overcome geographical barriers to care.

## Provenance statement

This article was not commissioned and went through external peer review.

## Reflexivity statement

This commentary was shaped by a diverse group of authors, ensuring a blend of perspectives. The primary author is a white European woman with extensive experience living in Rwanda. Her insights are enhanced by two Rwandan authors working at the Ministry of Health. Further authors consist of a Rwandan woman, an Italian woman and a Nigerian man, broadening the cultural and geographic scope. The team's expertise is predominantly medical, with five authors having backgrounds as doctors, a nurse and a midwife, including degrees in public health. These authors are complemented by the sixth author's background in international law.
